# Characteristics of real-world driving behavior in people with schizophrenia: a naturalistic study utilizing drive recorders

**DOI:** 10.1038/s41537-025-00613-1

**Published:** 2025-04-18

**Authors:** Hiroki Okada, Saki Komagata, Mayu Takagi, Yuichi Kamata, Junichi Matsumoto, Takaya Maeyama, Yukiko Takashio, Masaki Matoba

**Affiliations:** 1 Department of Rehabilitation of Sciences, Hokkaido University, Sapporo, Hokkaido Japan; 2 Medical Corporation Muroi Hospital, Ootawara, Tochigi Japan; 3 Social Welfare Service Corporation Bois de Boulogne, Sano, Tochigi Japan; 4 Social Welfare Service Corporation Hamagiku, Hitachinaka, Ibaraki Japan; 5 Graduate School of Health Sciences, Hokkaido University, Sapporo Hokkaido, Japan; 6 Medical Corporation Fukuroda Hospital, Daigo, Ibaraki Japan

**Keywords:** Schizophrenia, Psychosis

## Abstract

Research on driving among people with schizophrenia (PWS) is limited to laboratory-based studies. The present study aimed to compare PWS with healthy controls (HC) in real-world driving behaviors using 500 km of driving data collected through dashcams. Neuropsychological tests were conducted to evaluate both PWS and HC. After the evaluation, we compared traffic violations with dangerous driving behaviors, such as sudden braking and abrupt steering. PWS were treated with antipsychotics and antiparkinsonians and were evaluated using the Positive and Negative Syndrome Scale (PANSS) and the Drug-Induced Extrapyramidal Symptoms Scale. Statistical analysis included factor analysis, which was employed to evaluate the classification of violations and driving behaviors; correlation coefficients were employed to evaluate actual driving behavior. The results indicated that the mean driving speed was significantly lower, instances of speeding were absent, and the frequency of inhibition-related driving violations, such as smartphone use while driving, was lower. Violations related to inattention were associated with sustained attention and the useful field of view (UFOV). These findings suggest that PWS are less likely to engage in reckless driving compared to HC. Furthermore, considering that driving violations among PWS may be attributed to cognitive errors, the results highlight the importance of assessing and addressing cognitive functions in driver assistance systems to promote safer driving. One limitation of the study was that the severity of psychopathology among the participants was relatively low, which may have contributed to the PANSS, its subscales, and factors being less predictive of traffic violations.

## Background

Driving is the primary mode of transportation in modern society, enabling the efficient movement of people and goods across both urban and rural areas^1^. The number of drivers is steadily increasing, including those with mental illnesses. A study by Brunnauer et al.^2^ found that 67% of individuals with mental illness hold a driver’s license, and 77% drive regularly. A significant portion of individuals with schizophrenia (hereafter referred to as PWS) also drive regularly^3^.

However, driving inherently presents various risks. According to the World Health Organization (WHO)^4^, global road fatalities continue to rise, with 1.35 million deaths annually and an additional 20–50 million individuals suffering non-fatal injuries or disabilities. Identifying risk factors for road accidents is crucial to ensure that all drivers, including those with mental disorders, can operate vehicles safely. Human factors contribute to 93% of all traffic accidents, with errors, violations, and deliberate risky behaviors as primary causes^5^. Therefore, accident prevention requires addressing more than just driver errors^6^.

Errors and violations arise from distinct mechanisms. Errors often reflect performance limitations, such as deficits in perception, attention, and information processing^7^. The difference between errors and violations parallels the distinction between driving skills and driving styles^8^. Understanding both driving-related errors and violations influenced by driving styles is essential for developing strategies to promote safe driving among PWS.

Despite the importance of this issue, research on driving behaviors in PWS remains limited. Most studies examining driving errors have relied on driving simulators in controlled settings. Wylie et al.^9^ found that PWS exhibited impaired steering accuracy, delayed braking reactions at red lights, and failures to stop. Similarly, St. Germain et al.^10^ reported that PWS struggled with lateral position control and tended to drive at lower speeds compared to healthy controls (HCs). However, other studies have reported conflicting findings. Fuermaier et al.^11^ found no significant differences in collision rates or responses to stimuli between PWS and HCs. Furthermore, Okada et al.^12^ compared real-world driving behaviors and found no significant differences in steering or braking responses between the groups. These inconsistencies persist, with reports indicating that only 10.7–32.5% of PWS possess the cognitive and psychomotor skills required for safe driving^13,14^. Simulator-based studies may fail to capture the specific characteristics of driving errors in PWS.

Beyond driving errors, investigating driving styles is critical, as driving risk is influenced by a combination of errors, violations, and intentional risky behaviors. Laboratory-based virtual environments do not accurately assess driving styles, highlighting the need for real-world driving observations. To date, no studies have directly evaluated real-world driving behaviors among PWS^15,16^. Objective tools such as driving recorders equipped with global positioning systems (GPS) and accelerometers effectively capture hazardous driving styles and situational errors in high-risk populations, including older adults and young drivers^17,18^. These devices offer valuable insights into accident risks among PWS by detecting speeding, sudden steering, and other violations in real-world driving contexts.

This study aims to clarify the driving styles of PWS and examine the relationships between their driving behaviors and characteristics, including symptoms and cognitive impairments. Prior research has established strong links between cognitive function and driving errors^19^. Existing systems often assume that PWS are at high risk for accidents, resulting in restrictions on their driving privileges. However, limiting driving without real-world assessment may be problematic. This study seeks to provide valuable insights into accident risks associated with schizophrenia, contributing to evidence-based recommendations for appropriate driving restrictions and the development of targeted support systems for safe driving.

The primary objective is to compare dangerous driving styles and traffic violations between PWS and HCs using driving records. The secondary objective is to investigate the relationships between dangerous driving styles, traffic violations, and PWS characteristics, including symptoms, cognitive impairments, and extrapyramidal symptoms.

## Methods

Participants were divided into two groups: PWS, according to the International Statistical Classification of Diseases and Related Health Problems, Tenth Revision (ICD-10), and HCs. Both groups were recruited from the suburban regions of Northern Kanto, Japan, an area known for its high rate of car usage due to an underdeveloped public transportation network^20^. Based on previous studies, our inclusion criteria were all participants aged 20–60 years who had resided in the study area for over a year, had at least 5 years of driving experience, and drove regularly (at least once a week)^12,21^. The sample size was determined via analysis, suggesting the recruitment of 19–27 participants, with group comparisons set at *p* < 0.05, power = 0.8, and an effect size of 0.5–0.6. Symptoms in PWS were assessed using the Positive and Negative Syndrome Scale (PANNS), with evaluations conducted by a trained clinical psychologist and an occupational therapist, both holding doctorates and expertise in symptom evaluation^22^. The severity of PWS symptoms was assessed using the total PANSS score, which is the sum of 3 subscales (Positive, Negative, and General Psychopathology).

The average intake of antipsychotic medications was calculated using chlorpromazine equivalence^23,24^. The Drug-Induced Extrapyramidal Symptoms Scale (DIEPSS) was employed to examine the relationship between the driving style of PWS and any extrapyramidal symptoms that might influence driving^25^. The DIEPSS, a structured tool used primarily in Japan, assesses the severity of extrapyramidal symptoms (EPS) associated with antipsychotic treatment. It includes 8 specific symptom—gait, bradykinesia, sialorrhea, muscle rigidity, tremor, akathisia, dystonia, and dyskinesia—rated on a scale from 0 (normal) to 4 (severe), with higher scores indicating greater impairment. No participants exhibited 1) substance use disorders, 2) neurological disorders, 3) intellectual disabilities, or 4) severe orthopedic conditions.

To evaluate driving-related cognitive functions, we assessed processing speed, sustained attention and inhibitory control, visual memory, and planning ability via the Trail-Making Test A (TMTA)^26^, Continuous Performance Test (CPT) go/no-go task^27^, Wechsler Memory Scale (WMS; visual recall)^28^, Zoo Map Test (ZMT)^29^, and Useful Visual Field of View (UFOV)^30^. These visual cognitive function tasks were selected based on their frequent use in previous studies examining the relationship between driving skills and cognitive function^30–32^. To enhance the generalizability of the findings, we used the Specific Level of Functioning Scale (SLOF) to measure overall social functioning in PWS^33^. SLOF scores ranged from 24–120, with higher scores indicating better social functioning. The scale comprised 3 domains: social, daily living, and employment^34^. This study was approved by the Ethics Committee of the Hokkaido University Graduate School of Health Sciences, and all participants provided written informed consent.

### Study instrument: The in-vehicle data recorder

**Fig. 1:**
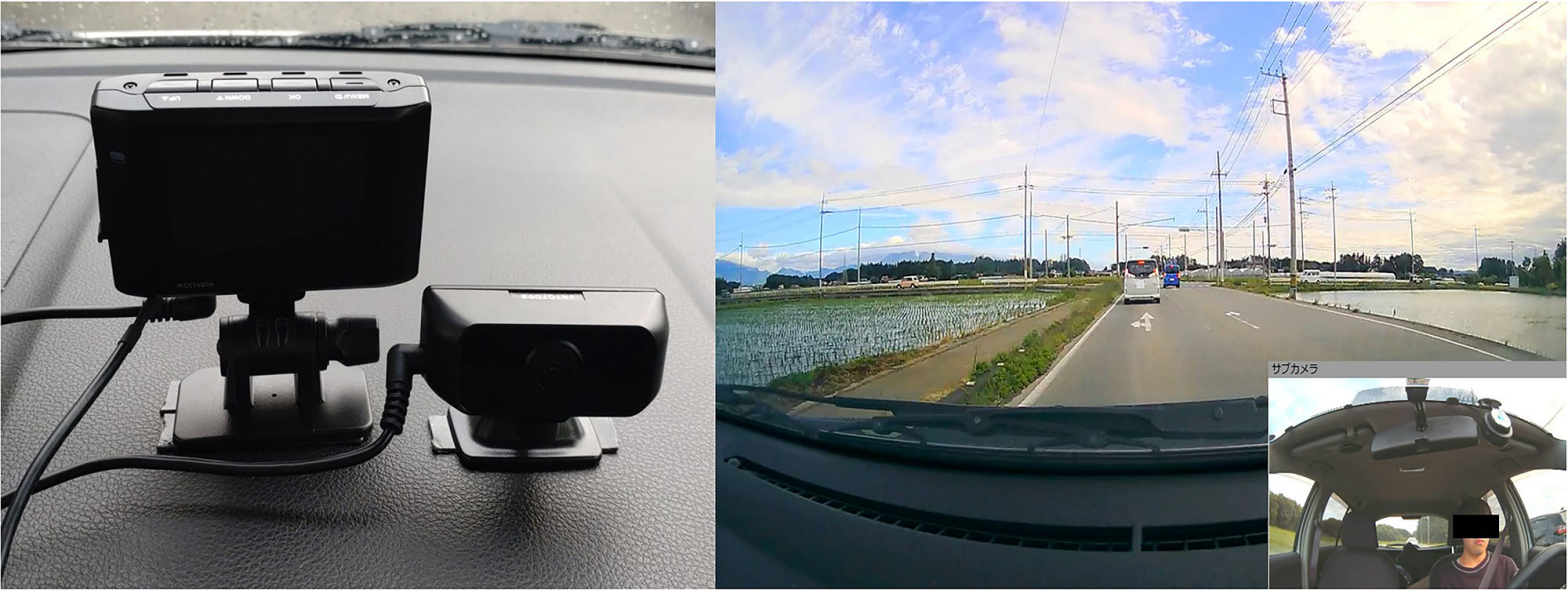
Drive Recorder and Sample Data Utilized in This Study. The figure on the left illustrates the drive recorder mounted on the dashboard of each vehicle used in the study. The figure on the right displays a representative example of the driving data recorded by the device.

To gather comprehensive and objective data on driving behavior, a drive recorder (HDR-953GW, COMTEC, Inc., Japan) was installed on the dashboard to record both the front and interior of the vehicle. Informed consent was obtained from participants (Fig. 1). The HDR-953GW continuously detected and recorded data while the vehicle was operating. Recorded data included vehicle speed, measured via GPS at 2 Hz, vehicle location, high acceleration events (such as sudden braking and sharp steering) detected via a three-axis accelerometer (10 Hz), forward-facing driving images from the front camera, and images of the driver’s behavior recorded by the in-vehicle camera.

### Measurement data

The analysis focused on the first 500 km of data collected by the drive recorder over 10 weeks. This inaugural study examined real-world driving behavior in PWS, so analysis was limited to participants’ routine driving activities. The analysis excluded (1) instances when someone other than the primary driver was in the vehicle, (2) driving outside the Northern Kanto region, 3) driving on expressways, and 4) trips exceeding 2 hours. Expressway driving and long-duration trips were excluded due to differing conditions from daily driving and the increased likelihood of destinations outside the regular driving area. Participants were also required to verify that they did not engage in drinking and driving during the 10-week period, a statement corroborated verbally by their family members or primary care workers.

Measured data included (1) average speed, (2) average maximum speed per trip, and 3) violation/dangerous driving behaviors, such as speeding (exceeding the limit by 20 km/h or more, and 30 km/h or more), running red lights, starting without observing traffic signals, ignoring stop signs, lane departures, failure to take proper safety precautions at intersections (e.g., illegal turns), and distracted driving (e.g., using a smartphone or operating a car navigation/audio system). Average speed, average maximum speed per trip, and violations involving sudden acceleration, steering, and braking were assessed via data from the drive recorder. Sudden acceleration, steering, and braking were defined as forces that exceeded 0.5 G. The remaining violations were analyzed per Japanese traffic laws, as outlined in Table 1. The first and second authors independently analyzed the recorded violations, and any discrepancies were resolved through discussion.

**Table 1:** Traffic Violations and Risky Driving Behaviors.

Violation	Traffic Violation Diagnostic Items
Speeding (exceeding by >20 km/h, >30 km/h)	Detected speeding violations (as measured by GPS)
Running red lights	Proceeding through a red light Accelerating to pass through an intersection during a signal transition
Starting without observing traffic signals	Initiating movement during the transition to a green signal
Ignoring stop signs	Failure to make a complete stop at intersections or designated areas controlled by a stop sign
Lane departures	Crossing the centerline and entering the oncoming traffic lane during vehicle operation
Traffic violations occurring within intersections	Improper stopping positions, incorrect turning techniques, obstruction of pedestrians, and interference with oncoming traffic during right turns
Distracted driving	Engagement in non-driving activities, such as smartphone usage, operating a navigation system, or adjustment of audio controls during vehicle operation.
Sudden acceleration	Acceleration exceeding 0.5 G as recorded by the accelerometer of the drive recorder
Sudden steering	Steering exceeding 0.5 G as recorded by the accelerometer of the drive recorder
Sudden braking	Braking exceeding 0.5 G as recorded by the accelerometer of the drive recorder

*GPS* global positioning system.

### Statistics

We used the Kolmogorov-Smirnov test to evaluate normality, and to compare cognitive function and basic demographic characteristics between the PWS and HC groups, a Mann-Whitney U test was conducted. Univariate general linear models (GLMs) compared average speed, average maximum speed per trip, and violations or risky driving behaviors between the groups. Variables with significant differences between the two groups were included as covariates in the GLM to control for baseline differences.

Factor analysis, using the unweighted least squares method with Promax rotation, identified latent factors related to violations and risky driving behaviors. Correlation analyses examined the relationships between each identified factor and variables such as cognitive function, schizophrenia-related characteristics (e.g., symptoms), mean speed, and maximum speed per trip. Partial correlations were computed, adjusting for variables that showed significant differences in the two-group comparison. Additionally, partial correlation analyses for the HC group examined associations between each factor and cognitive function, mean speed, and maximum speed per trip.

Finally, multiple regression analysis was used to interpret the significant variables identified in the partial correlation analyses.

## Results

The participant characteristics are summarized as follows: (a) significant differences were observed only in educational background between PWS and HC; (b) PWS had lower cognitive performance than HC, particularly in TMTA, CPT reaction time, WMS, ZMT, and UFOV; and (c) most PWS on antipsychotic medication, with 90% (*n* = 18) prescribed atypical antipsychotics, 10% (*n* = 2) receiving typical antipsychotics, and 5% (*n* = 1) on a combination of both. Additionally, 35% (*n* = 7) were undergoing polypharmacy with atypical antipsychotics, and 55% (*n* = 11) received antiparkinsonian medication for extrapyramidal symptoms. (Supplementary Table 1).

Table 2 presents the Mann-Whitney U test results for demographic attributes and cognitive function. The table provides details on the use of antipsychotic and antiparkinsonian medications in PWS. In summary, all PWS participants were in remission, on adequate doses of antipsychotics, and some required antiparkinsonian medication to manage prominent extrapyramidal symptoms.

**Table 2:** Demographics and Clinical Details of People with Schizophrenia and Healthy Controls.

Variable	PWS (*N* = 20)	HC (*N* = 20)	Statistics (Z)	*p*
Age	46.3 ± 11.1 (43)	43.6 ± 13.5 (38.5)	−0.921	0.357
Male (%)	12 (55)	12 (55)	0.000^※^	1.000
Education (years)	12.5 ± 1.9 (12)	14.5 ± 1.8 (16)	−3.116	**0.002**
Driving experience (years)	17.6 ± 8.6 (19.5)	18.9 ± 11.5 (17.5)	-0.095	0.924
Parking tickets in the past two years (number)	0 ± 0 (0)	0 ± 0 (0)	0.000	1.000
Accidents in the past two years (number)	0.1 ± 0.3 (0)	0 ± 0 (0)	0.000	1.000
Duration of illness (years)	20.9 ± 4.3 (21)	None		
Chlorpromazine equivalent (mg)	591.7 ± 277 (600)	None		
Atypical antipsychotics dose (mg)	566.3 ± 297 (600)	None		
Typical antipsychotic dose (mg)	537.5 ± 194 (537)	None		
Trail making test A (s)	50.1 ± 13.8 (47)	35.7 ± 10.5 (35.5)	−3.669	**0.000**
Continuous performance test				
Correct answer rate (%)	94.5 ± 8.6 (95)	96.8 ± 2.6 (95.2)	−1.468	0.121
Reaction time (ms)	531.3 ± 110.4 (500)	495.8 ± 96.4 (480)	−2.311	**0.021**
Coefficient of variation	14.3 ± 4.3 (13)	13.9 ± 4.2 (13.9)	−0.117	0.871
Wechsler memory test (visual memory I)	27.2 ± 8.5 (29)	35.5 ± 4.1 (36.5)	−3.720	**0.000**
Zoo map tests	9.7 ± 3.2 (9)	13.1 ± 3.7 (16)	−2.609	**0.009**
Useful field of view	43.9 ± 7.4 (46)	46.5 ± 6.1 (48)	−2.334	**0.020**
PANSS ^a)^ total	48.1 ± 6.2 (49)	None		
PANSS positive	12.8 ± 3.5 (12)	None		
PANSS negative	12.1 ± 3.6 (12)	None		
PANSS general psychopathology	23.2 ± 3.6 (23)	None		
DIEPSS ^b)^	5.2 ± 3.2 (5)	None		
Specific Levels of Functioning Scale	98.1 ± 6.6 (97)	None		

Numbers represent the mean ± standard deviation (median); *PWS* people with schizophrenia; *HC* healthy control.

※χ^2^ value; a) PANSS: Positive and Negative Syndrome Scale; b) Drug-Induced Extrapyramidal Symptoms Scale. Bold values indicate statistical significance.

### Differences in violations/driving styles between PWS and HC

The main findings of this analysis are as follows: (a) PWS exhibited lower average and maximum speeds per drive compared to HC, even after adjusting for covariates such as education, TMTA, CPT reaction time, WMS, ZMT, and UFOV; (b) PWS were less likely to engage in speeding violations; and (c) PWS showed less tendency to engage in distracted driving, such as smartphone use. Table 3 presents the results of the univariate GLM analysis for these driving parameters, measured across 500 km, as well as violations and dangerous driving behaviors.

**Table 3:** Comparison of Traffic Violations and Risky Driving Behaviors of People with Schizophrenia and Healthy Controls.

Variable	PWS (*N* = 20)	HC (*N* = 20)	*F*-value	*p*
Average speed (km/h)	34.7 ± 5.3 (34)	39.5 ± 4.5 (39)	**4.339**	**0.045**
Average maximum speed per trip (km/h)	62.6 ± 6.9 (60.5)	72.1 ± 7.6 (71)	**11.904**	**0.000**
Speeding (>20 km/h; km)	6.3 ± 8.7 (3)	18.5 ± 1.4 (15.5)	**10.13**	**0.003**
Speeding (>30 km/h; km)	0.2 ± 0.5 (0)	15.7 ± 6.7 (15)	**5.072**	**0.031**
Running red lights	6.9 ± 4.7 (6.5)	6.5 ± 3.9 (5)	0.732	0.399
Starting without observing traffic signals	0.00 ± 0.00 (0)	0.00 ± .00 (0)	0.000	1.000
Ignoring stop signs	24.1 ± 22.7 (17)	18 ± 10.5 (18)	0.339	0.738
Lane departures	1.4 ± 2.6 (1)	18 ± 10.5 (18)	1.009	0.324
Traffic violations occurring within intersections	0.7 ± 1.3 (1)	1.8 ± 3.2 (1)	0.481	0.493
Distracted driving	0.4 ± 0.7 (1)	31.5 ± 14.8 (27.4)	**6.893**	**0.013**
Sudden acceleration	0.0 ± 0.0 (0)	0.0 ± 0.0 (0)	0.000	1.000
Sudden maneuvers	1.8 ± 3.1 (1)	9.6 ± 21.1 (1)	0.437	0.518
Sudden braking	11.1 ± 20.9 (5)	5.3 ± 6.7 (3)	0.732	0.399

Numbers represent the mean ± standard deviation (median); *PWS* people with schizophrenia, *HC* healthy control, Covariates Education, TMTA, CPT: Reaction time, WMS, ZMT, and UFOV. Bold values indicate statistical significance.

### Analysis of factors behind violations/dangerous driving styles

The findings are as follows: (a) factor analysis identified three distinct categories of violations—speed violations, inattention violations, and inhibition violations—while sudden braking was excluded from this classification; (b) speed violations were correlated with average maximum speed per trip, and inattention violations were linked to the UFOV; (c) sudden braking in PWS was associated with extrapyramidal symptoms, as measured by the DIEPSS, rather than speeding behavior; and (d) Furthermore, in partial correlation analyses, inattention violations and sudden braking were found to be significantly associated with attentional function, as measured by the CPT, suggesting that these types of violations in PWS may be particularly linked to underlying cognitive impairments.

Table 4 presents the factor analysis results, categorizing violations based on characteristics. Tables 5–8 show the correlation analysis and multiple regression analysis results between these factors and cognitive function, and other PWS characteristics, as well as average and maximum speed per trip via partial correlation analysis. Control variables included education, TMTA, CPT reaction time, WMS, ZMT, and UFOV, consistent with the univariate GLM model.

**Table 4:** Factor Analysis of Violations and Dangerous Driving Behaviors (*N* = 40).

Variable	Eigenvalue	Factor Loadings
Factor 1 (Speed violation)	2.636	
Speeding (>20 km/h)		0.607
Speeding (>30 km/h)		0.990
Sudden steering		0.842
Factor 2 (Inattention violation)	2.061	
Running red lights		0.518
Ignoring stop signs		0.438
Lane departures		0.757
Traffic violations occurring within intersections		0.624
Factor 3 (Inhibition violation)	1.169	
Distracted driving		0.950
No factor	None	
Sudden braking ^a)^		–

a) Factor Loadings of Sudden braking: Factor 1; .213; Factor 2; −0.027; Factor 3; −0.128.

**Table 5:** Correlation Analysis of the Factors Behind Violations/Dangerous Driving Styles of People with Schizophrenia (N = 20).

Variable	TMTA	CPT:C ^a)^	CPT:R	CPT:C ^b)^	WMS	ZMT	UFOV	DIEPS	CPZeq	Average speed	Average maximum speed per trip
Factor 1 (Speed violation)	−0.335	−0.390	0.401	0.323	−0.013	−0.118	−0.241	0.057	0.153	0.382	0.570*
Factor 2 (Inattention violation)	−0.458	−0.237	−0.042	−0.640*	−0.041	−0.270	−0.545*	−0.143	−0.260	−0.436	−0.418
Factor 3 (Inhibition violation)	0.270	0.029	0.290	−0.214	0.226	0.007	0.100	0.294	0.103	−0.344	−0.138
sudden braking	−0.204	−0.715**	0.177	0.421	−0.272	0.263	0.261	0.637*	0.126	−0.300	0.230

*<0.05; **<0.01.

Factor 1: Speeding (>20 km/h), Speeding (>30 km/h), and Sudden steering.

Factor 2: Running red lights, Ignoring stop signs, Lane departures, and Traffic violations occurring within intersections.

Factor 3: Distracted driving.

Trail making test A: TMT; (a) Continuous performance test: Correct answer rate: CPT:C; Continuous performance test: Reaction time: CPT:R; (b) Continuous performance test: Coefficient of variation CPT:C; Wechsler memory test (visual memory I): WMS; Zoo map tests: ZMT; Useful field of view: UFOV; Drug-Induced Extrapyramidal Symptoms Scale: DIEPS; Chlorpromazine equivalent: CPZeq.

Control variables: Education, TMTA, CPT:Reaction time, WMS, ZMT, and UFOV. Bold values indicate statistical significance.

**Table 6:** Correlation Analysis of the Factors behind Violations/Dangerous Driving Styles of Healthy Controls (*N* = 20).

Variable	TMTA	CPT:C ^a)^	CPT:R	CPT:C ^b)^	WMS	ZMT	UFOV	Average speed	Average maximum speed per trip
Factor 1 (Speed violation)	0.104	−0.221	−0.265	0.087	0.325	−0.475	−0.174	0.264	0.868**
Factor 2 (Inattention violation)	−0.263	−0.080	0.190	−0.333	−0.200	0.189	0.053	−0.401	0.341
Factor 3 (Inhibition violation)	−0.315	−0.414	−0.242	0.360	0.169	0.328	0.147	−0.135	−0.274
Sudden braking	0.282	−0.297	−0.020	0.406	0.274	−0.486	−0.163	0.474	0.618*

*<0.05; **<0.01.

Factor 1: Speeding (>20 km/h), Speeding (>30 km/h), and Sudden steering.

Factor 2: Running red lights, Ignoring stop signs, Lane departures, and Traffic violations occurring within intersections.

Factor 3: Distracted driving.

Trail making test A: TMTA; (a) Continuous performance test: Correct answer rate: CPT:C; Continuous performance test: Reaction time: CPT:R; (b) Continuous performance test: Coefficient of variation: CPT:C; Wechsler memory test (visual memory I): WMS; Zoo map tests: ZMT; Useful field of view UFOV.

Control variables: Education, TMTA, CPT: Reaction time, WMS, ZMT, and UFOV. Bold values indicate statistical significance.

**Table 7:** Multiple Regression Analysis of the Factors Behind Violations/Dangerous Driving Styles of People with Schizophrenia (N = 20).

Dependent Variable	Independent Variable	β (SE)	95% CI	*p*-value
Factor 1^a)^ (Speed violation)	Average maximum speed per trip	1.663 (0.582)	0.436–2.886	**0.01**
Factor 2^b)^ (Inattention violation)	Continuous performance test: Reaction time	−83.928 (68.8)	−22.16–9.344	0.56
	Useful field of view	−1.315 (0.788)	−3.472–0.144	**0.03**
Sudden braking^c)^	Continuous performance test: Correct answer rate	−9.04 (6.832)	−23.02–5.399	0.203
	Drug-Induced Extrapyramidal Symptoms Scale	3.528 (1.424)	0.522–0.653	**0.024**

Factor 1: Speeding (>20 km/h), Speeding (>30 km/h), and Sudden steering.

Factor 2: Running red lights, ignoring stop signs, lane departures, and traffic violations occurring within intersections.

(a) Adjusted R²; .272 F-statistic; 8.125, (b) Adjusted R²; .198 F-statistic; 3.335, (c) Adjusted R²;.201 F-statistic; 7.327. Bold values indicate statistical significance.

**Table 8:** Multiple Regression Analysis of the Factors Behind Violations/Dangerous Driving Styles of Healthy Controls (N = 20).

Dependent Variable	Independent Variable	β (SE)	95% CI	*p*-value
Factor 1^a)^ (Speed violation)	Average maximum speed per trip	5.382 (0.698)	3.923–6.842	**<0.001**
Sudden braking ^b)^	Average maximum speed per trip	0.033 (0.01)	0.011–0.0544	**0.004**

Factor 1: Speeding (>20 km/h), Speeding (>30 km/h), and Sudden steering.

Factor 3: Distracted driving.

(a) Adjusted R²; .756, F-statistic;60.05, Adjusted R²; .338 F-statistic; 10.73. Bold values indicate statistical significance.

To determine whether sudden braking was a response to near-miss incidents, the context of each braking event was analyzed. Results showed that 36% of sudden braking incidents occurred at traffic lights, 21% at intersections, 33% to maintain distance from the vehicle ahead, and 10% to avoid obstacles. Most incidents were deemed unnecessary, reinforcing that sudden braking in PWS was not related to speeding. A more detailed analysis of symptoms found no significant association between schizophrenia-related symptoms and violations.

In contrast, in HC, cognitive function was not correlated with violations, while both speeding and sudden braking were associated with the average maximum speed per day.

Partial correlation analyses were conducted to examine the relationship between symptoms. The results are presented in Supplementary Table 2. Notably, no significant association was found between symptoms and schizophrenia violations.

## Discussion

The real-world driving data from this study showed that PWS tended to avoid secondary tasks while driving and maintained a slow driving speed. The authors believe the results are consistent with the simulator studies, and suggests that, by adopting compensatory behaivors^11^, PWS drive cautiously to avoid accidents in the real world^11,35^. Recent simulator studies have indicated that individuals with schizophrenia often exhibit cognitive impairments and may be only partially fit to drive^36^. However, the present study found that they compensate for these impairments by avoiding speeding and some traffic violations. Thus, the real-world discovery that PWS actively attempt to drive more safely has important implications for supporting driving and promoting social inclusion among this population.

Furthermore, multiple regression analysis revealed that PWS exhibited different braking patterns compared to HC, with no significant correlation between abrupt braking and maximum driving speed. Most abrupt braking incidents were not responses to near-miss accidents but occurred during routine driving maneuvers, such as stopping at intersections or traffic signals. Exploratory analysis identified an association between abrupt braking and extrapyramidal symptoms. Braking requires an immediate response to stimuli, and motor impairments in PWS may hinder smooth execution, leading to excessive braking. The fact that many participants were taking anti-parkinsonian medications further supports this link to motor control difficulties. In most cases, abrupt braking occurred during routine stopping maneuvers at red lights or intersections. Motor impairments, such as coordination deficits and postural control issues, affected even those treated with atypical antipsychotics, including the participants in this study^37^.

Another notable finding was that, unlike HC, the violations committed by PWS appeared to be associated with cognitive vulnerabilities. Specifically, the UFOV was linked to inattention-related violations, such as running red lights or failing to stop at stop signs. UFOV measures the visual field from which a driver can gather information without moving their head or eyes. A narrower UFOV increases the likelihood of missing visual cues, elevating the risk of traffic signal and sign violations. The correlation between reduced UFOV and impaired driving performance has been well-documented in older drivers^38^. In PWS, declines in visual processing speed and peripheral vision recognition may contribute to missed traffic signs and red-light violations.

Regarding inhibitory violations, such as smartphone use while driving, no significant correlations were found with the variables measured in this study. This may be because most PWS participants did not engage in such violations.

Finally, no significant correlations were observed between cognitive function and the four types of violations in HC. This suggests that the violations committed by HC may be more closely related to personality traits rather than cognitive impairments^39^.

## Limitations

This study has several limitations. First, the PANSS, along with its subscales and factors, would likely not be predictors of traffic violations, as patients exhibited a low degree of psychopathology severity. Moreover, our study focused solely on daily driving, in which participants drove alone and were confined to suburban areas. Future studies should examine different conditions, such as driving with passengers, in urban areas or over long distances. Furthermore, this study, one of the first to analyze driving in real-world settings for PWS, examined various variables without adjusting for significance levels. However, considering that many items revealed moderate or higher correlations, especially in the correlation analysis, we believe that the effect size was reliably achieved.

## Conclusion

Despite its limitations, this study identified distinct differences in driving styles between PWS and HC. Violations and dangerous driving behaviors in PWS may be linked to cognitive function. Therefore, two key propositions can be made. First, the assessment of cognitive function plays a crucial role in driving assistance, as it may predict certain traffic violations and risky driving behaviors. Additionally, we recommend developing cognitive functional training to enhance safe driving^40^. Current cognitive function training primarily targets improvements in daily life and work-related tasks, with no research specifically focusing on driving. Enhancing cognitive functions related to sustained attention and UFOV may be beneficial for maintaining concentration, complying with traffic signals, and stopping appropriately. Furthermore, unnecessary sudden braking in PWS can be mitigated by considering extrapyramidal symptoms when prescribing medications. Driving assistance that accounts for these specific characteristics is essential to help PWS achieve greater mobility and support their recovery.

## Supplementary information

Supplementary Table (Characteristics of Antiparkinsonian Medication Use, and Symptoms, and Traffic Violations or Risky Driving Behaviors of People with Schizophrenia)

## Data Availability

Data supporting the findings of this study are available from the corresponding author upon reasonable request.
