# Supplementary material for: Characteristics of real-world driving behavior in people with schizophrenia: a naturalistic study utilizing drive recorders
Source: Schizophrenia (Heidelb). 2025 Apr 18;11(1):67. doi: 10.1038/s41537-025-00613-1 (PMC12006520; doi:10.1038/s41537-025-00613-1)
Supplement: Supplementary file 1 — Supplementary Table (Characteristics of Antiparkinsonian Medication Use, and Symptoms, and Traffic Violations or Risky Driving Behaviors of People with Schizophrenia) [file 41537_2025_613_MOESM1_ESM.docx]

| **Supplementary Table 1: Antiparkinsonian medications used** (n=11) | | |
| --- | --- | --- |
|  | Individuals receiving medication | |
| Biperiden | 8 |  |
| Bromocriptine | 1 |  |
| Pramipexole | 1 |  |
| Trihexyphenidyl | 2 |  |
| **One individual is concurrently taking both Pramipexole and Trihexyphenidyl** | | |

| **Supplementary Table 2: Correlation analysis of the factors behind violations/dangerous driving styles of people with schizophrenia** **(N=20)** | | | | |
| --- | --- | --- | --- | --- |
|  | PANSS ^a)^ total | PANSS positive | PANSS negative | PANSS general psychopathology |
| Factor 1  (Speed violation) | -.341 | .117 | -.489 | -.187 |
| Factor 2  (Inattention violation) | -.035 | -.269 | .419 | -.222 |
| Factor 3  (Inhibition violation) | -.143 | -.409 | .505 | -.354 |
| sudden braking | -.190 | .012 | -.227 | -.099 |
| Factor 1: Speeding (>20 km/h), speeding (>30 km/h), and sudden steering  Factor 2: Running red lights, ignoring stop signs, lane departures, and traffic violations occurring within intersections  Factor 3: Distracted driving  a) PANSS (Positive and Negative Syndrome Scale) | | | | |
